# Glucocorticoid induced adrenal insufficiency in children: Morning cortisol values to avoid LDSST

**DOI:** 10.3389/fped.2022.981765

**Published:** 2022-12-15

**Authors:** Margaux Laulhé, Cécile Dumaine, Didier Chevenne, Fallou Leye, Albert Faye, Blandine Dozières, Marion Strullu, Jérome Viala, Julien Hogan, Véronique Houdouin, Juliane Léger, Dominique Simon, Jean-Claude Carel, Caroline Storey, Sophie Guilmin-Crépon, Laetitia Martinerie

**Affiliations:** ^1^ Pediatric Endocrinology Department, AP-HP, Reference Center for Growth and Development Endocrine Diseases Hôpital Universitaire Robert-Debré, Paris, France; ^2^ Université Paris-Saclay, Inserm 1185, Physiologie et Physiopathologie Endocriniennes, Le Kremlin-Bicêtre, France; ^3^ General Pediatrics and Infectious Diseases Department, AP-HP, Hôpital Universitaire Robert-Debré, Paris, France; ^4^ Biochemistry Unit, AP-HP, Hôpital Universitaire Robert-Debré, Paris, France; ^5^ Clinical Epidemiology Unit, AP-HP, Hôpital Universitaire Robert-Debré, Paris, France; ^6^ UFR Médecine, Université Paris Cité, Paris, France; ^7^ Pediatric Neurology Department, AP-HP, Hôpital Universitaire Robert-Debré, Paris, France; ^8^ Pediatric Hematology and Immunology Department, AP-HP, Hôpital Universitaire Robert-Debré, Paris, France; ^9^ Pediatric Gastroenterology and Hepatology Department, AP-HP, Hôpital Universitaire Robert-Debré, Paris, France; ^10^ Pediatric Nephrology Department, AP-HP, Hôpital Universitaire Robert-Debré, Paris, France; ^11^ Pediatric Pulmonology and Allergology Department, AP-HP, Hôpital Universitaire Robert-Debré, Paris, France; ^12^ Université Paris Cité, Inserm 1141, NeuroDiderot, Paris, France

**Keywords:** adrenal insufficency, low dose short synacthen test, glucocorticoid withdrawal, cortisol, pediatric

## Abstract

**Objectives:**

Glucocorticoid-induced adrenal insufficiency (GI-AI) is a common side effect of glucocorticoid therapy. However, its diagnosis currently relies on the realization of a Low Dose Short Synacthen Test (LD-SST) that requires an outpatient hospital and several blood samples. Our goal was to evaluate whether morning cortisol values could predict the response to LD-SST, in children, to avoid useless dynamic tests and facilitate diagnosis of glucocorticoid induced adrenal insufficiency.

**Study Design:**

We recorded data of 91 pediatric patients who underwent a LD-SST in our center between 2016 and 2020 in a retrospective observational study. We selected LD-SST realized following administration of supra-physiologic doses of glucocorticoids during more than 3 weeks and performed at least four weeks after treatment was stopped. Adrenal deficiency was defined as a plasma cortisol concentration inferior to 500 nmol/l at LD-SST.

**Results:**

Glucocorticoid-induced adrenal insufficiency was diagnosed in 60% of our cohort. Morning cortisol values were predictive of the response to the LD-SST (AUC ROC 0.78). A plasma cortisol concentration of less than 144 nmol/l predicted glucocorticoid induced adrenal insufficiency with a specificity of 94% and a value over 317 nmol/l predicted recovery of the HPA axis with a sensitivity of 95%. We did not find any other predictive factor for glucocorticoid-induced adrenal insufficiency.

**Conclusions:**

Morning cortisol values can safely assess recovery of the HPA axis in children treated chronically with glucocorticoids. Using these thresholds, more than 50% of LD-SST could be avoided in children.

## Introduction

Glucocorticoids are widely used for their anti-inflammatory and immunosuppressive properties. Depending on the study, glucocorticoids users represent between 0.5% and 20% of the population (1–4), with approximately 1% with chronic use (1, 3). In pediatrics, glucocorticoids are commonly used in asthma (5), eczema (6), inflammatory diseases such as Crohn disease (7) or arthritis (8) and in acute leukemia (9) or Graft vs. Host disease (GvHD) (10) treatment. Numerous side effects may be associated such as diabetes, growth retardation or glucocorticoid-induced adrenal insufficiency (GI-AI) (11, 12).

Synthetic glucocorticoids are derived from cortisol, an endogenous hormone, synthesized in the zona fasciculata of the adrenal cortex (13). Cortisol is an essential regulator of homeostasis on resting and stress conditions, through its action on immune system, skeletal muscles, central nervous system, glucose and electrolytic homeostasis. Cortisol secretion is regulated by the AdrenoCorticoTropin Hormone (ACTH), synthesized in the pituitary gland, secretion of which is controlled by the Corticotropin Releasing Hormone (CRH), secreted by the paraventricular nucleus of the hypothalamus (14). Cortisol exerts negative feedback on the biosynthesis of CRH and ACTH.

Chronic exposure to extra-physiologic concentrations of glucocorticoids causes a decrease in ACTH stimulation on the adrenal gland and can eventually lead to its atrophy with an inability of the HPA axis to respond to an acute stress (15).

Prevalence of GI-AI varies between 13% and 63% during the first month of glucocorticoids withdrawal (16), with persistence after 3 years for 15% of the patients. This variability may be partially explained by the heterogeneity in the synthetic glucocorticoids prescribed, in the duration of treatment or the modality of evaluation. However, GI-AI is the most important side effect of glucocorticoids and is still underdiagnosed because of the lack of specific symptoms.

Nevertheless, several cases of acute adrenal crises have been described and in a retrospective Dutch study, authors found that adrenal crises are more frequent in patients with GI-AI compared to patients with other secondary adrenal insufficiencies (17–20). GI-AI is a severe but avoidable complication of glucocorticoids use.

Diagnosis of central adrenal insufficiency is based on a dynamic evaluation of the HPA axis. The gold standard is the insulin tolerance test (ITT) but it is not commonly used in the pediatric population and is even prohibited in young infants because of the significant risk of hypoglycemic brain damage (21). The low-dose (1 µg) Short Synacthen Test (LD-SST), which has shown similar efficacy to ITT (22) is the most frequent test used in pediatrics because of fewer contraindications and risk of complications.

LD-SST is not without consequences, both at the medical and socio-economic level, as it requires an outpatient hospital, a peripheral venous line, and multiple blood samples. In order to limit the use of a dynamic test to evaluate GI-AI after prolonged glucocorticoids therapy, some authors have tried to define a threshold of morning cortisol (12, 23, 24) or ACTH (25) value that would be sufficient to conclude to a normal corticotropic function. However, data in pediatric population is lacking.

In this study, we aimed to assess whether morning plasma cortisol was associated with the response to LD-SST in children and identify thresholds of morning cortisol values that would predict with great accuracy the response of the HPA axis to the LD-SST, thus defining conditions where this test would not be necessary. Our secondary objectives were to determine if cut-off values of ACTH could also be predictive of the response to the LD-SST and to evaluate potential predictive factors of GI-AI.

## Patients and methods

### Patients

Data of all pediatric patients (between 6 months old and 18 years old) who underwent a LD-SST in a tertiary pediatric hospital in Paris, France from January 2016 to December 2020 were collected retrospectively.

Inclusion criteria were:

– patients who had taken chronic glucocorticoid treatment, defined as a dose of at least 12 mg/m^2^/day (hydrocortisone) or 3 mg/m^2^/day (prednisone) during at least 3 weeks according to the literature (23, 26).
– patients who had stopped their glucocorticoids treatment, except for replacement therapy, at least 4 weeks before the realization of the test (27).

Exclusion criteria were:

– patients who underwent the LD-SST for suspicion of secondary adrenal insufficiency due to pituitary tumors, radiotherapy or ectopic pituitary gland
– patients still under glucocorticoid treatment (aside from replacement therapy)

If more than one LD-SST was performed for the same patient, only the first LD-SST was considered.

This retrospective observational study was approved by the Institutional Review Board (IRB 00006477, N 2020-529) and was conducted according to the General Data Protection Regulation (GDRP).

### Low Dose-Short Synacthen Test

The LD-SST was performed by trained nurses, dedicated to endocrine tests, in the morning between 8 and 9 am, in the Pediatric Outpatient Department Unit, according to the protocol in use at our hospital.

At *t* = 0, plasma basal cortisol and ACTH concentrations were sampled. ACTH was immediately carried on ice to the Biochemistry Unit. Subsequently, a 1 µg bolus of synthetic ACTH (28) (Synacthen) was administered intravenously freshly prepared by diluting a 0.25 mg/ml tetracosactide (Novartis Pharma) ampulla in 249 ml of normal saline. After 20 min, plasma cortisol was sampled again (T20).

Normal response to the LD-SST was defined by an elevation of plasma cortisol level after the 1 µg Synacthen injection of more than 500 nmol/L (29).

### Biological analyses

Cortisol hormonal assessments were performed by the Biochemistry Department of the hospital using immunoassays. Techniques evolved in February 2019. Prior, hormonal dosages were measured using the Centaur CP Immunoassay System, Siemens; the intra and inter-assays coefficients of variation were <4.8% and <6.5% respectively. From this date and onward, cortisol was assessed using ACCESS 2 automated immunoassay (DXi-BeckmanCoulter) with intra and inter-assays coefficients of variation <4.6% and <6.4% respectively. It implied a modification in sensitivity. Thus, for result harmonization in the study, values were converted. A conversion factor (= 1/slope) was extracted from the slope of a simple linear regression model comparing 34 plasma cortisol values analyzed with each of the two devices. We converted our values according to the formula: ancient cortisol = new cortisol/0.757.

ACTH dosage was performed using immunoradiometric assay (IRMA) until April 2020 (ELSA-ACTH, Cis-Bio); the intra and inter-assays coefficients of variation were <6.5% and <15% respectively. From this date and onwards, ACTH concentration was assessed with immunoluminometric assay (CLIA) using LIAISON ACTH (Liaison XL-Diasorin), the intra and inter-assays coefficients of variation were <4% and <7.5% respectively. This modification did not lead to a significant modification of ACTH values.

### Data collection

Data were collected from computerized medical records: birth date and assigned gender, the date and results of the LD-SST (cortisol at T0 and T20 in nmol/L), basal ACTH concentration (ng/L) if available, dates of onset and arrest of glucocorticoid treatment, weight (kg) and height (cm) at the initiation of glucocorticoid treatment, also expressed as SDS for age and sex, the different types of glucocorticoids administrated including intra-venous or per os, topical, inhalation and the maximal dose of oral or intra-venous glucocorticoid received (expressed in prednisone equivalent dose).

We also collected existence of glucocorticoid related complications.

All the data were collected and processed with the Epidata v4.6.0.2 software.

### Data analyses

Statistical analyses were performed by the investigators, with the support of the clinical epidemiology unit of the hospital.

Qualitative variables are described as numbers and percentages; quantitative variables are expressed by median [Q1; Q3].

The search for a predictive threshold was carried out using ROC curves.

The search for factors associated with glucocorticoid-induced adrenal insufficiency was performed by a logistic regression model, with the integration of significant explanatory variables at the threshold of 0.20 for univariate analyses. The number of explanatory variables was defined to meet the conditions of application of the test (1 explanatory variable for 10 events).

Statistical analyses were performed using SAS 9.4 software.

## Results

### Characteristics of the population

**Figure 1:**
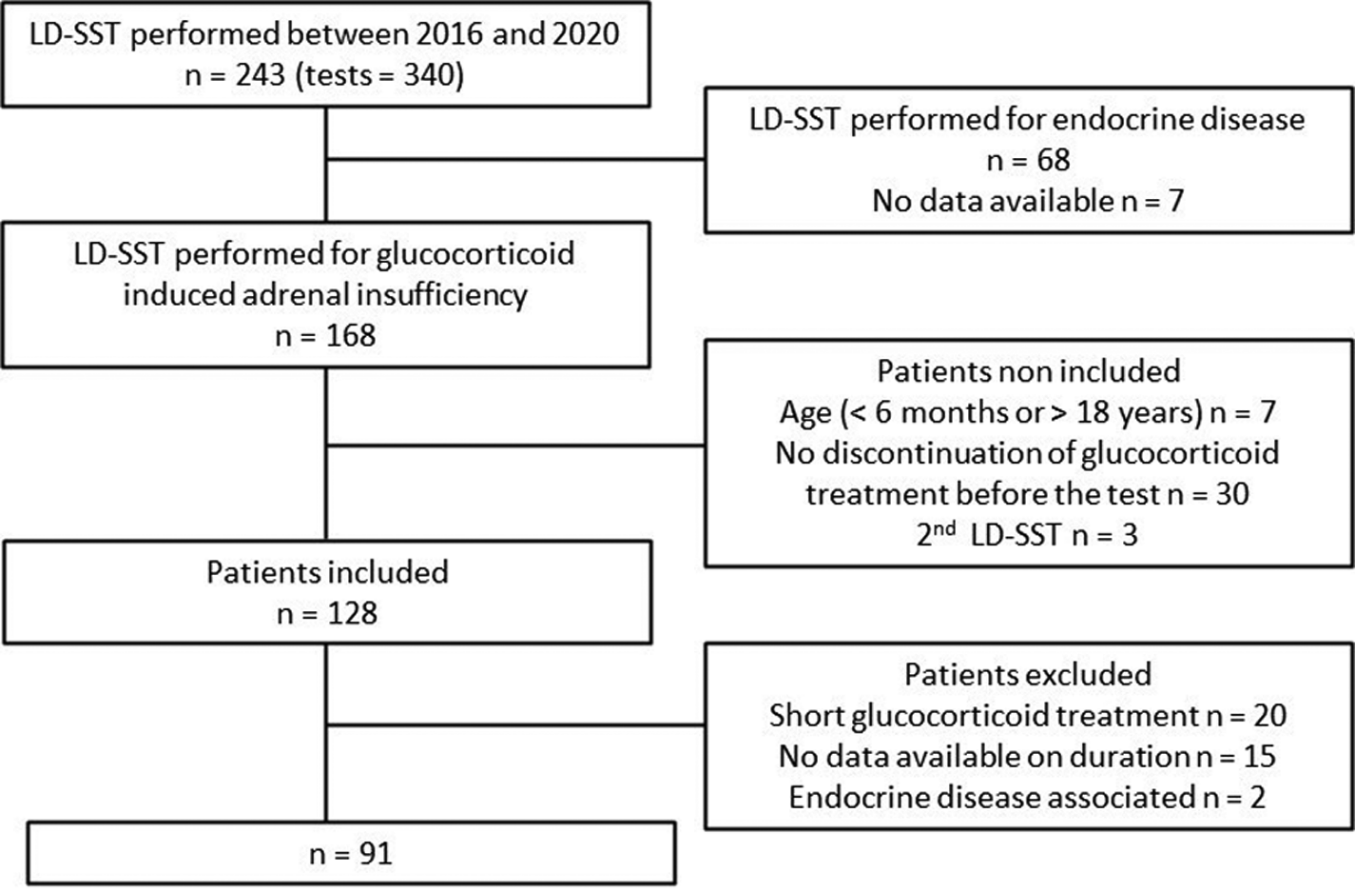
Flow chart of the study.

Between January 2016 and December 2020, 340 LD-SSTs have been performed in the hospital for 243 patients (Figure 1**)**. Only patients who had been explored for suspicion of GI-AI (*n* = 168) were included. Patients older than 18 years old or younger than 6 months old at the realization of the test (*n* = 7), patients who had not stopped glucocorticoid treatment at least 4 weeks before the test (*n* = 30) or patients with a previous LD-SST performed in a different center (*n* = 3) were excluded. Among the remaining 128 patients those who had not been exposed chronically to glucocorticoids (*n* = 20) were excluded. We also excluded patients who presented endocrine disease that may have interfered with the results of the test (central GH insufficiency suggesting hypopituitarism, *n* = 2). At last, 91 tests were analyzed, due to 15 patients with missing data on the duration of the treatment.

Clinical characteristics of the population are presented in Table 1. Children were treated for a median time of 5.2 months, with an onset of treatment at a median age of 7 years old. The duration of treatment was calculated from the first to the last day of administration preceding LD-SST. Almost 50% of the population was treated for inflammatory disease and approximately 20% for Graft vs. Host Disease (GvHD) and epileptic spasms. The small percentage of patients treated for asthma included in the study is explained by the continuous use of inhaled glucocorticoids by the time the LD-SST was performed for most asthmatic patients, which induced their exclusion from the study. All patients (*n* = 91) were treated with oral glucocorticoids. Forty-three patients (47%) were treated with intravenous glucocorticoids before oral treatment.

**Table 1:** Population characteristics.

	Distribution *N* = 91
**Sex**	
Girl	46 (50.5%)
**Pathology**	
Asthma	1 (1%)
GvHD	17 (19%)
Inflammatory disease (IBD, AJI, uveitis, …)	45 (49.5%)
Epileptic spasms	19 (21%)
Others^a^	9 (10%)
**Glucocorticoid treatment**	
Age at the onset of treatment (years)	7.0 [1.3;11.2]
Duration of the treatment (months)	5.2 [2.48;11.93]
Delay between arrest of glucocorticoids and LD-SST (months)	6.0 [3.0;13.6]
**Number of glucocorticoid administration modes**	
1	59 (65%)
>1	32 (35%)
**Administration route associated with oral treatment**	
IV	43 (47%)
Cutaneous	16 (18%)
Inhaled	21 (23%)
Other topic administration	8 (9%)
**Complications associated with glucocorticoids**	
At least 1 complication	32 (35%)
HBP	21 (66%)
Bone disease^b^	4 (12.5%)
Glucocorticoid-induced diabetes	5 (16%)
Other complications^c^	14 (44%)
**Associated treatment interfering with the HPA axis**	13 (14%)

Data are expressed as means (%) for qualitative values and medians [Q1;Q3] for continuous variables.

^a^ Other pathologies are hemolytic and uremic syndrome (*n* = 1), tracheal stenosis (*n* = 1), lung disease apart from asthma (*n* = 2), dermatologic disease apart from eczema (*n* = 2), hematologic disease (*n* = 2), post-infectious inflammation of the aorta and intra-cranial hypertension (*n* = 1).

^b^ Bone diseases include aseptic osteonecrosis (*n* = 1) and osteopenia (*n* = 3).

^c^ Other complications include cushing syndrome (*n* = 3), purple striae (*n* = 6), hypoglycemia (*n* = 1), psychiatric disease (*n* = 3) and infection (*n* = 1). GvHD: Graft versus Host Disease, IBD: Inflammatory Bowel Disease, AJI: Acute Juvenile Arthritis, HBP: High Blood Pressure.

More than 30% of the patients received more than one glucocorticoid treatment at a time. Most patients were treated with prednisone. Treatments that might have interfered with the HPA axis: imidazole anti-fungus treatment, total body irradiation or phenytoin, were administrated with glucocorticoids for almost 15% of the patients. At least 1 complication, related to glucocorticoids, was described for 32 patients. The most frequent side effect was high blood pressure which concerned 23% of the population (*n* = 21), then glucocorticoid-induced diabetes for 15% of the children. Three children presented with psychiatric disorders including one patient who required anxiolytic treatment.

### Morning plasma cortisol level, unlike ACTH, is associated with the response LD ld-SST

**Figure 2:**
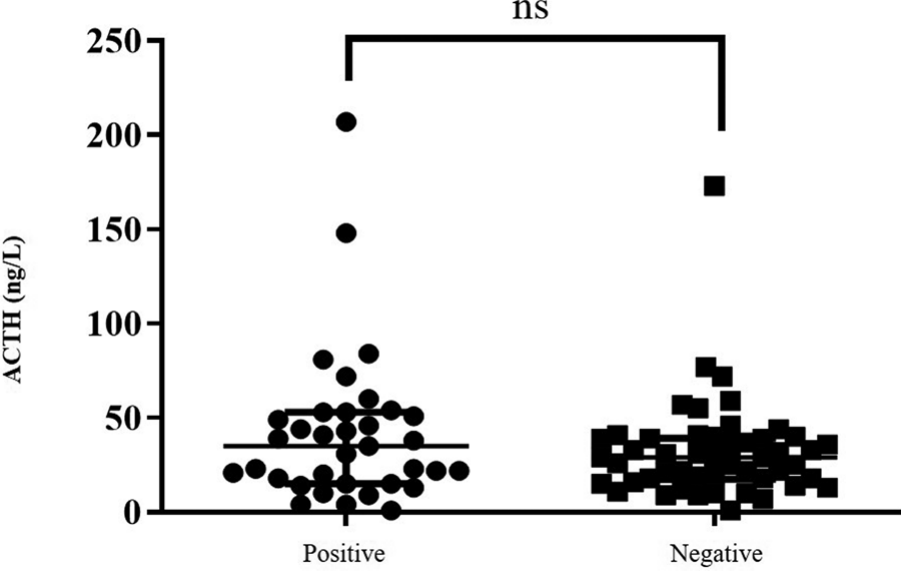
Morning plasma ACTH levels according to the result of the LD-SST. Distribution of ACTH dosages is represented with median and interquartile range depending on the results of the LD-SST. LD-SST was considered positive when cortisol at T20 raised above 500 nmol/L otherwise it was considered negative. Association between ACTH and GI-AI did not reach statistical significance using Analysis Of Variance (ANOVA, *p* = 0.18, ns).

**Figure 3:**
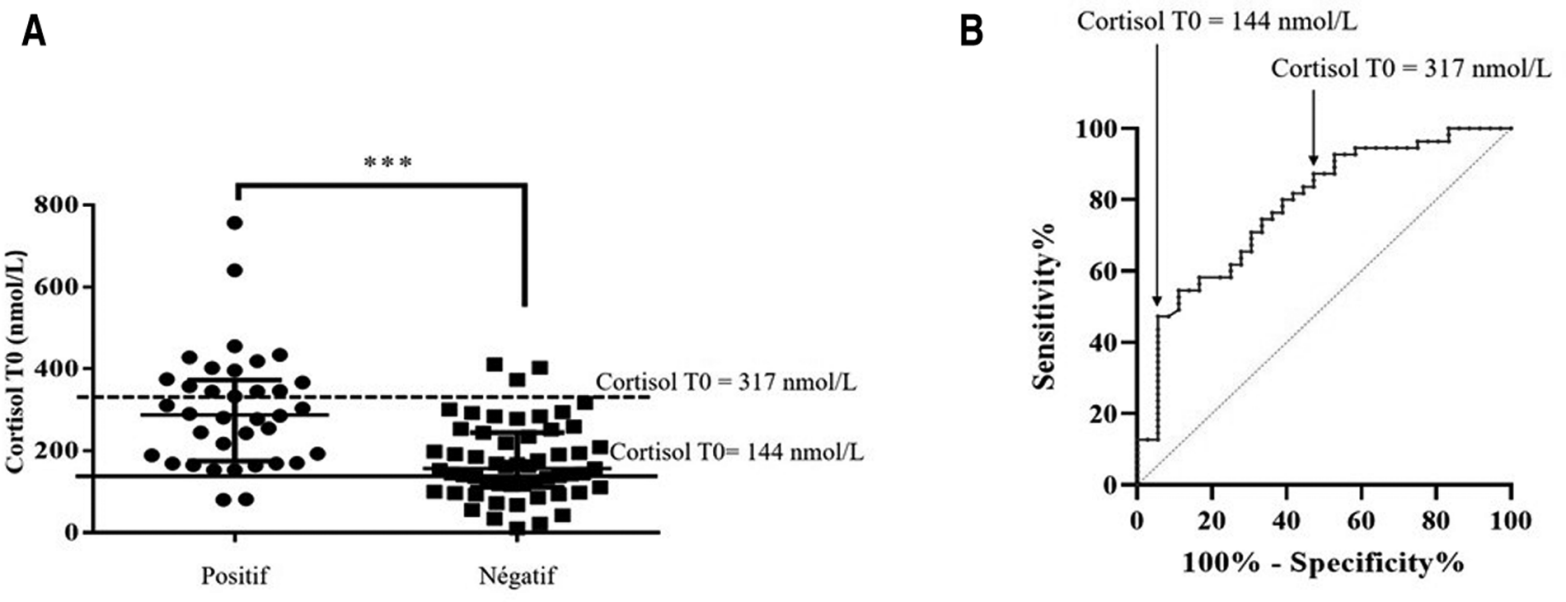
Morning plasma cortisol levels according to the result of the LD-SST (A) morning plasma cortisol value was associated to the LD-SST (ANOVA, ***p < 0.0001). LD-SST was considered positive when cortisol at T20 raised above 500 nmol/l otherwise it was considered negative. The full line represents the value associated with GI-AI (Se = 0.47, Sp = 0.94). The dotted line represents the value associated with a Normal response to LD-SST (Se = 0.95, Sp = 0.42). **B)** ROC curve for morning plasma cortisol level with LD-SST as the reference test. Area under the curve is 0.7795 (IC95 [0.69; 0.88], *p* value < 0.001).

In our cohort, 55 patients (60%) were diagnosed with GI-AI (assessed by a plasma cortisol concentration inferior to 500 nmol/L at T20 of the LD-SST). ACTH levels were recorded for 85 patients. No association was observed between basal ACTH levels and GI-AI (Figure 2, *p* = 0.18, online only). However, we found a significant association between morning plasma cortisol value, collected at the beginning of the test, and LD-SST response at T20 (Figure 3A, *p* < 0.0001).

### A morning plasma cortisol value of less than 144 nmol/L or superior to 317 nmol/L, predicts the results of the LD-SST

Since morning cortisol was associated with LD-SST outcome (Figure 3A), we analyzed morning plasma cortisol levels sensitivity and specificity to predict GI-AI using a ROC curve. The area under the curve was 0.7795 (IC95 [0.6826; 0.8765]; *p* < 0.0001, Figure 3B). A morning plasma cortisol value inferior to 144 nmol/L predicted adrenal insufficiency with a specificity of 94% (Figure 3B). Negative predictive value was 0.92 (Table 2). Using this threshold value, only two patients (2.2%) in our cohort would be misclassified and unnecessarily treated.

**Table 2:** Frequencies of test outcome depending on the LD-SST result.

	LD-SST results					
Cortisol T0 (nmol/L)	Positive (cortisol T20 > 500 nmol/L)	Negative (cortisol T20 < 500 nmol/L)	Sensitivity	Specificity	Positive predictive value	Negative predictive value
>317 nmol/L	15	3	95%	42%	0.83	NA
<144 nmol/L	2	24	42%	94%	NA	0.92

As we could not predict response to LD-SST if the morning cortisol value is in-between the two thresholds chosen, we could not extract a negative predictive value for the upper cut-off (317 nmol/L) or a positive predictive value for the lower cut-off (144 nmol/L) chosen.

On the other hand, if plasma cortisol concentration is superior to 317 nmol/L, our model predicts a positive response to the test with a sensitivity of 95% (Figure 3B). Positive predictive value was 0.83 (Table 2).

However, this would imply that 3 patients (3.3%) would be considered to have normal adrenal response while they require a replacement therapy according to the standards of the test.

### Factors associated with glucocorticoid-induced adrenal insufficiency

We analyzed the association between GI-AI and sex, age, duration of treatment, other glucocorticoid-induced complications and number of glucocorticoid treatments (Table 3).

**Table 3:** Analyses of potential predictive factors for HPA axis recovery.

Variable	OR [IC 95%]	*P* value
**Sex**		
Boys	1	
Girls	1.68 [1.02;2.707]	0.23
**Age at onset of treatment**		
[0.5;2]	1	
|2;10]	0.77 [0.28;2.1]	0.62
>10	0.78 [0.26;2.31]	0.63
**Number of concomitant glucocorticoid treatments**		
1	1	
>1	1.21 [0.72;2.01]	0.67
**Growth rate during the first year of treatment (SDS)**	1.06 [1.00;1.11]	0.26
**Associated treatment**		
No	1	
Yes	1.37 [0.69;2.74]	0.6
**Other glucocorticoid induced complications**		
No	1	
Yes	0.65 [0.33;1.27]	0.46
**Treatment duration**	** **	** **
[0;0.5]	1	
[0.5;1]	1.05 [0.56;1.98]	0.92
>1	0.77 [0.42;1.42]	0.62
**Delay between arrest of treatment and LD-SST**		
	0.99 [0.96;1.03]	0.93

Association between clinical features and HPA axis recovery was analyzed using a linear regression model. Age at the onset of treatment is separated in three categories: infants between 6 months and 2 years old (0.5–2), children between 2 and 10 years old (2–10) and adolescents of more than 10 years (>10). Treatment duration is divided in 3 categories: less than 6 months (0.5), between 6 months and a year (0.5–1) and more than a year (>1).

A positive growth rate during the first year of treatment and female sex seem to be associated with absence of GI-AI, although not with statistical significance.

The duration of treatment, the presence of glucocorticoid-induced complications and the age at onset of treatment were not significantly associated with GI-AI.

As none of the factors studied reached a statistical significance of 20%, we did not perform additional multivariate analysis.

### Discussion

LD-SST performed in our outpatient clinic during the last 4 years for suspicion of GI-AI were analyzed to assess the correlation between morning cortisol values and GI-AI in children and to define thresholds of morning cortisol that would predict or rule out GI-AI. The final objective was to evaluate if, thus, unnecessary LD-SST could be potentially avoided.

This study reports a large well-characterized pediatric cohort who underwent a LD-SST for suspicion of GI-AI. To our knowledge, this is one of the largest pediatric cohort studied evaluating thresholds of morning cortisol predicting the results of LD-SST following chronic glucocorticoid treatment (23, 30). The main limitation of our study is its retrospective design which may have led to collection bias and thus the thresholds should be confirmed in a prospective study. Furthermore, interpretation of morning cortisol thresholds may vary depending on the assay used, thus our results should be interpreted taking this into account (31).

A recent meta-analysis defines an absolute risk of GI-AI of 48.7% (32). The prevalence of GI-AI varies depending on the delay between arrest of treatment and the realization of the LD-SST but is approximately 40% between 10 weeks and 1 year after cessation of treatment (16). In a pediatric retrospective study, GI-AI was diagnosed in 42% of the patients evaluated with LD-SST performed between 1 and 66 days after glucocorticoid withdrawal (23). It appears that performing a LD-SST too early after glucocorticoids withdrawal or while ongoing therapy still exceeds 3 mg/m^2^/day (equivalent prednisone) may lead to misinterpretation and unnecessary repetition of the tests as shown by the high prevalence of GI-AI during early days after withdrawal (27). In our pediatric population for whom the LD-SST was performed mostly around 6 months after glucocorticoids arrest, 60% of the patients were diagnosed with GI-AI. It could be explained by a recruitment bias in our cohort. Indeed, patients who had had chronic glucocorticoid treatment but were not systematically explored for GI-AI were not included, thus selecting those with higher doses or longer use of glucocorticoid therapy. Prevalence of GI-AI should thus be further evaluated in a prospective study including all patients who underwent a chronic glucocorticoid treatment of more than 3 weeks at a dose of at least 3 mg/m^2^ equivalent prednisone.

In our hospital, among the 243 patients who underwent a LD-SST, 168 were subjected to it for suspicion of GI-AI (the other LD-SSTs were performed for suspicion of endocrine diseases like combined pituitary hormonal deficiency). Patients explored for suspicion of GI-AI represent more than 65% of all the LD-SST realized in the hospital during this period and support the idea that GI-AI is an important matter of children health (33).

We analyzed results from 91 LD-SST over the 168 realized in our center, using more stringent selection criteria than those usually found. Particularly, we excluded patients that still had ongoing supraphysiological doses of glucocorticoids. Thus, our population differs from others studies considering the types of pathologies presented by the children, in the sense that asthma and allergy, which are the most common diseases for which glucocorticoids are used, were scarcely represented (23, 34, 35). However, GI-AI is a real issue for asthmatic patients, a recent study from Zöllner et al., shows that GI-AI is found in more than 50% of children with inhaled glucocorticoids treatment (33).

Considering these 91 LD-SST, our study confirms that morning plasma cortisol concentrations are associated with response to LD-SST in children. This was previously demonstrated for adult patients (36, 37). However, as morning cortisol values are considered based on cortisol values at the beginning of the test, it would be interesting to test whether this correlation is also accurate if the test is performed at another time during the day. To our knowledge, only one pediatric study has evaluated the association between serum baseline cortisol and LD-SST but did not assess thresholds to predict cortisol response to LD-SST (23).

We found that a morning cortisol (at the beginning of the test) value inferior to 144 nmol/L, predicted glucocorticoid-induced adrenal insufficiency with a specificity of 94%. In addition, a morning cortisol value superior to 317 nmol/L, predicted recovery of the HPA axis with a sensitivity of 95% Thus, using these thresholds, approximately 50% of LD-SST (*n* = 49) could have been avoided.

A morning cortisol cut-off value of 144 nmol/L to assess GI-AI is consistent with the threshold of 138 nmol/L defined by a meta-analysis including children (28, 38), whereas the 317 nmol/L cut-off is lower than those established in adults. Considering the pediatric population, a retrospective study including 31 patients with suspicion of central adrenal insufficiency evaluated by a CRH test and 22 healthy controls found that morning cortisol was associated with the result of the test (30). Thresholds for dynamic tests and 9 am cortisol plasma levels were defined according to the 10th percentile of the values obtained in healthy controls. Interestingly, the cut-off value was 140 nmol/L. Furthermore, a morning plasma cortisol level superior to 289 nmol/L predicted a functional HPA axis with a sensitivity of 96% and a specificity of 50%, which is very close to our results.

In our study, a cut-off value for morning plasma cortisol level of 317 nmol/L resulted in the misclassification of 3 patients. Nevertheless, the peak of plasma cortisol at T20 was superior to 480 nmol/L for all three patients, thus raising interrogation concerning the cut-off value for the LD-SST. The 500 nmol/L cut-off value used in our center is the most frequently used (23, 39), however, in the literature 450 nmol/L and 400 nmol/L cut-off values are discussed (40, 41). Using salivary free cortisol, Vaiani et al., defined a new cut-off value for LD-SST of 450 nmol/L, which can discriminate intermediate insufficiency from real insufficiency to avoid unnecessary hormone replacement. Interestingly, considering a cut-off value of 450 nmol/L, none of our patients would have been misclassified (40).

In addition, the response of the HPA axis was evaluated in our center regarding the rise in cortisol at T20. Considering that after stimulation, cortisol usually reaches a maximum after 30 min, our LD-SST could underestimate the potency to reach the 500 nmol/L cut-off (30, 42).

Finally, our study did not highlight any predictive factors for GI-AI. Duration of treatment and cumulative dose in relation to GI-AI are debated in the literature (43, 44), however, no strong predictive factor for GI-AI has emerged. In fact, individual variation in glucocorticoid sensitivity, as genetic factors, prevents any lower limits of dose or duration or administration type that would safely rule out any risk of GI-AI (45). Potential individual or external predictive factors should thus be evaluated prospectively.

## Conclusion

We found that using morning plasma cortisol values can safely avoid realization of LD-SST for 50% of the patients with a suspicion of GI-AI. According to the peak value of plasma cortisol used in our center, a morning cortisol lower than 144 nmol/L indicates the need to continue the replacement therapy whereas a cortisol higher than 317 nmol/L allows to stop replacement therapy. For patients with a morning plasma cortisol value between 144 and 317 nmol/L, a LD-SST may be discussed, or replacement therapy should be pursued with a new morning plasma cortisol value assessed after a few weeks.

## Data Availability

The raw data supporting the conclusions of this article will be made available by the authors, without undue reservation.
